# Characterization of Extended-Spectrum β-Lactamase-Producing *Enterobacteriaceae* From Retail Food in China

**DOI:** 10.3389/fmicb.2018.01709

**Published:** 2018-08-08

**Authors:** Qinghua Ye, Qingping Wu, Shuhong Zhang, Jumei Zhang, Guangzhu Yang, Juan Wang, Liang Xue, Moutong Chen

**Affiliations:** ^1^State Key Laboratory of Applied Microbiology Southern China, Guangdong Provincial Key Laboratory of Microbial Culture Collection and Application, Guangdong Open Laboratory of Applied Microbiology, Guangdong Institute of Microbiology, Guangzhou, China; ^2^College of Food Science, South China Agricultural University, Guangzhou, China

**Keywords:** *Enterobacteriaceae*, extended spectrum β-lactamase, antimicrobial resistance, β-lactamase, retail food, China

## Abstract

In this study, we characterized the β-lactamase genes and phenotypic resistance of cephalosporin-resistant *Enterobacteriaceae* isolated from retail foods in China. Of 1,024 *Enterobacteriaceae* isolates recovered from raw meat products, aquatic products, raw vegetables, retail-level ready-to-eat (RTE) foods, frozen foods, and mushrooms from 2011 to 2014, 164 (16.0%) showed cefotaxime (CTX) and/or ceftazidime (CAZ) cephalosporin resistance, and 96 (9.4%) showed the extended-spectrum β-lactamase (ESBL) phenotype. More than 30% isolates were resistant to all antimicrobial agents except carbapenems (MEM 3.1% and IPM 5.2%), cefoxitin (FOX 6.3%), and amoxicillin/clavulanic acid (AMC 26%), and 94.8% of the strains were resistant to up to seven antibiotics. Polymerase chain reaction analysis showed that blaTEM (81.9%) was the most common gene, followed by blaCTX-M (68.1%) and blaSHV (38.9%). Moreover, 16.8% (72/429) of food samples contained ESBL-positive *Enterobacteriaceae*, with the following patterns: 32.9% (23/70) in frozen foods, 27.2% (5/29) in mushrooms, 17.6% (24/131) in raw meats, 13.3% (4/30) in fresh vegetables, 11.1% (8/72) in RTE foods, and 9.3% (9/97) in aquatic products. In addition, 24 of 217 foods collected in South China (11.1%), 25 of 131 foods collected in North of the Yangtze River region (19.1%), and 23 of 81 foods collected in South of the Yangtze River region (28.4%) were positive for ESBL- *Enterobacteriaceae*. Conjugation experiments demonstrated that the 22 of 72 isolates were transconjugants that had received the β-lactamase gene and were resistant to β-lactam antibiotics as well as some non-β-lactam antibiotics. These findings demonstrated that retail foods may be reservoirs for the dissemination of β-lactam antibiotics and that resistance genes could be transmitted to humans through the food chain; and the predominant ESBL-producing *Enterobacteriaceae* in China was isolated from in frozen chicken-meat, followed by frozen pork, cold noodles in sauce, cucumber, raw chicken meat, frozen pasta, brine-soaked chicken and tomato.

## Introduction

*Enterobacteriaceae*, a large family of gram-negative, non-spore-forming, rod-shaped, facultative anaerobes capable of fermenting sugars to various end products, are important hygiene indicators for process verification in food production, recently replacing the poorly defined group of coliforms (Anonymous, 2005). Several members of this group, including some species of *Escherichia, Enterobacter, Klebsiella, Proteus, Citrobacter, Serratia, Salmonella, Shigella*, and *Yersinia*, are among the most important causes of serious hospital-acquired and community-onset bacterial infections in humans (Jarzab et al., 2011).

Resistance to antimicrobial agents in *Enterobacteriaceae* has become an increasingly relevant problem. Because antibiotics are widely used in medical clinics and animal husbandry, high concentrations of antibiotics have appeared in aquacultures and other agricultural products, soil, water, and even food; consistent with this, antimicrobial-resistant *Enterobacteriaceae* have been appeared more and more frequently, and multiple drug-resistant strains have emerged (Capita and Alonso-Calleja, 2013; Laxminarayan et al., 2013). Beta-lactams (mainly extended-spectrum cephalosporins and carbapenems) and fluoroquinolones are the primary therapeutic choices to treat infections caused by *Enterobacteriaceae* (Bassetti et al., 2015). However, resistance to these antimicrobials has been increasing in recent years (Ben Sallem et al., 2012; Blaak et al., 2014; Durso and Cook, 2014).

β-Lactamases are the most frequent source of resistance to β-lactam antibiotics, and the production of β-lactamase is the primary mechanism of antibiotic resistance in *Enterobacteriaceae*. Various β-lactamases have been reported, including penicillinases, extended-spectrum β-lactamases (ESBLs), cephalosporinases (AmpC), metallo-β-lactamases (MBLs), and carbapenemases (KPCs) (Chagas et al., 2011; Pitout, 2012). Among these, ESBL-producing bacteria are particularly important (Bush and Jacoby, 2010; Nordmann et al., 2011). ESBLs are mainly produced in gram-negative bacilli, particularly *Enterobacteriaceae* (Poirel et al., 2012), including the well-known bacteria *Escherichia coli* and *Klebsiella pneumoniae* (Cantón et al., 2012; Poirel et al., 2012). ESBLs have the ability to hydrolyze penicillins and aztreonam, as well as first-, second-, and third-generation cephalosporins, but not cephamycins and carbapenems. Moreover, ESBLs are usually inhibited by so-called “classical” β-lactamase inhibitors, such as clavulanic acid, tazobactam, and sulbactam (Lee et al., 2012). Most ESBLs can also confer resistance to fourth-generation cephalosporins [such as cefepime (FEP) or cefpirome] (Bush and Jacoby, 2010). ESBLs include the classical extended-spectrum TEM-, SHV-, OXA-, and cefotaxime (CTX)-M type enzymes (Livermore, 2008; Pfeiier et al., 2010).

Previous studies have focused on investigating ESBLs in medical and veterinary clinics; however, relatively few reports have investigated ESBLs in foods (Reuland et al., 2014; Ben Said et al., 2015; Tekiner and Özpinar, 2016). Because of the wide use of broad-spectrum antibiotics in the livestock and animal husbandry industries, ESBL-producing bacteria have evolved and shown increased incidence owing to mutations, selection, and the spread of drug-resistant genes in animal and food products of animal origin (Ojer-Usoz et al., 2013; Tekiner and Özpinar, 2016), as well as other types of foods, such as seafood (Nguyen et al., 2016), raw vegetables (Reuland et al., 2014; Ben Said et al., 2015), ready-to-eat (RTE) foods (Campos et al., 2015; Kim et al., 2015), and milk (Odenthal et al., 2016). Furthermore, these ESBL bacteria can readily be transferred to humans through consumption of contaminated food, contributing to the spread and persistence of antibiotic-resistant bacteria in the general population and environment (Huijbers et al., 2016). Thus, in order to identify changes in antimicrobial resistance as early as possible and control the spread of ESBL-producing strains in foods, implementing a system for monitoring the prevalence patterns of ESBL-producing *Enterobacteriaceae* in foods from different areas and performing regular surveillance of the susceptibility of isolates to antimicrobial agents are necessary.

In China, most studies of ESBL-producing *Enterobacteriaceae* have focused on characterizing isolates from the clinical setting (Xiao et al., 2011, 2015), aquatic environment (Zou et al., 2012), animals (Wang et al., 2012; Bai et al., 2016), and animal food sources (Zheng et al., 2012; Yang et al., 2014) in particular areas or provinces. In these studies, only limited types of food samples were examined. No systematic studies have been conducted on a nationwide scale in China.

Accordingly, based on our prior research on the prevalence of *Enterobacteriaceae* in certain cities (Yang et al., 2016; Zhang et al., 2016), we undertook this study with the goal of providing new knowledge on the diversity of *Enterobacteriaceae* from different types of fresh food, frozen food and retail-level RTE food samples collected from 2011 to 2014, covering most provincial capitals of China, characterize their resistance to antibiotics, and determine the presence of ESBL- producers in the recovered isolates.

## Materials and methods

### Bacterial strains

A total of 1,024 *Enterobacteriaceae* strains were studied; these strains were isolated from 429 retail food samples (including raw meat products, aquatic products, raw vegetables, retail-level RTE foods, frozen foods, and mushrooms) from various cities in China from 2011 to 2014 (Table S1). The strains were selected from the collection of the Guangdong Institute of Microbiology in Guangzhou, China. In brief, for isolation of *E. coli*, 25 g food sample was placed into a sterile bag containing 225 mL Butterfield's phosphate-buffered water (Huankai, Guangzhou, China) and homogenized at 230 rpm for 2 min using a stomacher (Huankai); serial 10-fold dilutions was inoculated lactose broth (Huankai) and fermentation tubes (Huankai) at 37°C for 24–48 h, a loopful of suspension from positive cultures (lactose fermentation positive and gas production positive) was streaked onto Chromagar *E. coli* agar plates (Huankai) and incubated at 37°C for 18–24 h; Subsequently, presumptive *E. coli* colonies were selected from each plate and biochemically identified by API 20E (BioMe′rieux, Marcy I′Etoile, France). For isolation of *Salmonella*, 25 g food sample was pre-enriched in 225 ml of buffered peptone broth (Huankai); one milliliter cultures were incubated in 10 ml of selenite cystine broth (SC) (Huankai) at 37°C and 10 ml of tetrathionate brilliant green broth (TTB) at 42°C for 24 h, respectively; loopfuls of SC and TTB cultures were streaked onto xyloselysine-tergitol 4 (XLT4) selective agar plates (Difco, Detroit, MI, USA) and chromogenic Salmonella agar plates (Huankai), then incubated at 37°C for 24 h; presumptive colonies were picked from each plate, stabbed into a triple sugar iron slant (Huankai), and incubated at 37°C for 24 h; isolates with typical Salmonella phenotypes were further confirmed using API 20E test strips (BioMe′rieux, Marcy I′Etoile, France).

### Phenotypic ESBL testing

Disk diffusion tests were used for initial screening of ESBL-producing isolates. Mueller Hinton agar (Huankai, Guangzhou, China) was swabbed using a suspension of a pure culture (0.5McF), and antibiotic disks were then loaded. CTX (30 μg) and ceftazidime (CAZ, 30 μg) were used for screening of suspicious ESBL-producing isolates (Maravić et al., 2015). Isolates with reduced susceptibility to CTX and/or CAZ were assessed for the presence of ESBLs using a combination of the double-disk synergy test (DDST) with CTX and CAZ with and without clavulanic acid (Mast Diagnostics, Merseyside, UK) according to the 2011 guidelines of the Clinical and Laboratory Standards Institute (CLSI). An increase in the zone diameter of more than 5 mm for either antimicrobial agent tested in combination with clavulanic acid vs. the zone diameter of the agent when tested alone confirmed the presence of an ESBL-producing organism.

### Antimicrobial susceptibility testing

The susceptibility of antimicrobials to other commonly used antibiotics was assessed using the disk diffusion method according to 2011 CLSI guidelines for the following antibiotics: ampicillin (AMP, 30 μg), amoxicillin/clavulanic acid (AMC, 20/10 μg), aztreonam (AZM, 30 μg), imipinem (IPM, 10 μg), meropenem (MEM, 10 μg), FEP (30 μg), gentamicin (CN, 10 μg), tobramycin (TM, 10 μg), tetracycline (TE, 30 μg), ciprofloxacin (CIP, 5 μg), levofloxacin (LEV, 5 μg), trimethroprim/sulfamethoxazole (SXT, 1.25/23.75 μg), sulfonamide (SSS, 300 μg), chloramphenicol (C, 30 μg), cephalothin (KF, 30 μg), cefoxitin (FOX, 30 μg), ceftiofur (EFT, 30 μg), CTX (30 μg), and CAZ (30 μg; all from Oxoid Ltd., Basingstoke, UK). *Staphylococcus aureus* ATCC 25923 and *Escherichia coli* ATCC 25922 were used as quality control strains for this study. Zones of inhibition were measured with a precision caliper to the nearest 0.01 mm. Isolates exhibiting resistance to at least three antimicrobial agents tested were considered as multidrug-resistant strains.

### Detection of beta-lactamase genes

All isolates that exhibited a positive phenotype in ESBL screening tests were evaluated by PCR for the presence of genes encoding TEM, SHV, OXA, and CTX-M as previously described, with minor modifications. Genomic DNA was extracted from ESBL-producing isolates using a Bacterial Genomic DNA Purification Kit (Dongsheng Biotech, Guangzhou, China) according to the manufacturer's instruction. Genomic DNA concentration was determined at 260 nm using a Nano Drop^®;^ND-1000UVeVis Spectrophotometer (Thermo Fisher Scientific, MA, USA). The primer sequences and their positions, PCR conditions, and references are summarized in Table S1.

### Detection of integrons

The presence of *intI1* and *intI2* genes (encoding class 1 and class 2 integrases, respectively) was examined by PCR (Table S1). Genomic DNA was extracted according to 2.4.

### Conjugation mating experiments

Conjugation experiments were performed with the plasmid-free recipient strain *E. coli* DH5α (Kallová et al., 1995). Briefly, single colonies of the donor and recipient were inoculated in LB broth (Huankai, Guangzhou, China) and grown overnight at 37°C. Subsequently, equal volumes of the donor and recipient cultures were mixed and incubated overnight at 37°C without shaking. Serial dilutions were then plated on Luria-Bertani agar (Huankai, Guangzhou, China) selection plates supplemented with 50 μg/mL AMP (National Institutes for Food and Drug Control, Beijing, China). For all transconjugation experiments, the donor strain alone and acceptor strain alone were used as controls to ensure the effectiveness of the selective plates used. Transconjugants growing on the selection plates were subjected to DDSTs and PCR to confirm the presence of the ESBL phenotype.

### Statistics

The independent sample Kruskal-Wallis test (multiple groups) were used to compare proportions of the resistances to antimicrobials for ESBL-producing strains between species, regions, and food types groups by SPSS Statistics V21.0; the independent sample Kruskal-Wallis test (multiple groups) were also used to compare proportions of ESBL-positive *Enterobacteriaceae* between food type and regions groups by SPSS Statistics V21.0.

## Results

### Identification of ESBL-producing *enterobacteriaceae*

A total of 164 (16.0%) isolates that showed the resistance or intermediate resistance to CTX and/or CAZ by the disc diffusion method were subjected to an initial screening of ESBL-producing isolates. Based on the results, 96 (9.4%) isolates were designated as ESBLs by DDSTs. Table 1 shows the identities of the 96 isolated ESBL-producing *Enterobacteriaceae*. A high prevalence of *E. coli* strains was found, followed by *Salmonella* spp. The remaining strains were identified as *Citrobacter freundii, C. koseri, C. amalonaticus*, and *Serratia marcescens*.

**Table 1:** Distribution of ESBL-producing *Enterobacteriaceae* isolates from retail foods in China.

**Species**	**Total isolated**	**Number of ESBL-producing strains (%)**	
		**CTX and CAZ-resistant isolates**	**Confirmed by DDST**
*E. coli*	547	112(20.5)	62(11.3)
*Salmonella* spp.	308	35(11.3)	26(8.4)
*Klebsiella* spp.	25	2(8.0)	0(0)
*K. pneumoniae*	9	2(22.2)	0(0)
*K. oxytoca*	14	0(0)	0(0)
*K. terrigena*	2	0(0)	0(0)
*Citrobacter* spp.	71	11(15.5)	7(9.9)
*C. freundii*	43	7(16.3)	4(9.3)
*C. koseri*	7	2(28.6)	1(14.3)
*C. amalonaticus*	9	2(22.2)	2(22.2)
*C. youngae*	4	0(0)	0(0)
*C. braakii*	8	0(0)	0(0)
*kluyvera* spp.	5	0(0)	0(0)
*Pantoea* spp.	4	1(25.0)	0(0)
*Hafnia alvei*	10	0(0)	0(0)
*Proteus vulgaris*	3	0(0)	0(0)
*Serratia* spp.	31	1(3.2)	1(3.2)
*S. liquefaciens*	7	0(0)	0(0)
*S. marcescens*	17	1(5.9)	1(5.9)
*S. odorifial*	1	0(0)	0(0)
*S. rubidaea*	1	0(0)	0(0)
*S. fonticola*	3	0(0)	0(0)
*S. ficaria*	2	0(0)	0(0)
*Cedecea davisae*	5	0(0)	0(0)
*Providencia Alcaligenes*	1	0(0)	0(0)
*Enterobacter* spp.	14	2(14.3)	0(0)
*E. cloacae*	7	1(14.3)	0(0)
*E. aegrogens*	3	1(33.3)	0(0)
*E. gergoviae*	2	0(0)	0(0)
*E. amnigenus1*	2	0(0)	0(0)
Total	1,024	164(16.0)	96(9.4)

### Sensitivity profiles of ESBL-producing strains

Antimicrobial susceptibility tests using the standard disk diffusion method confirmed the presence of 96 ESBL-producing strains from DDSTs. The β-lactam sensitivity patterns showed high resistances to AMP, CTX, KF, and EFT (97.9, 97.8, 96.9, and 95.8%, respectively). The remaining sensitivity patterns were as follows: AZM, 51.0% resistant and 22.9% intermediate; CAZ, 42.7% resistant and 19.8% intermediate; FEP, 30.2% resistant and 17.7% intermediate; AMC, 26% resistant and 45.8% intermediate; and FOX, 6.3% resistant and 93.7% sensitive. The resistance against carbapenems was low; approximately 5.2% and 5.2% of strains showed resistance and intermediate resistance to IPM and MEM, respectively. Additionally, resistance patterns were observed for SSS (92.7% resistant and 2.1% intermediate), SXT (81.3% resistant), TE (83.3% resistant and 1.1% intermediate), and C (83.3% resistant and 2.1% intermediate). In contrast, approximately 36.5–47.9% of the strains were sensitive to CN, TM, LEV, and CIP (47.9.8, 40.6, 37.5, and 36.5%, respectively; Table 2). In addition, with the exception of one strain that was resistant to only one antimicrobials and one strain that was resistant to two antibiotic, all strains exhibited a multidrug-resistant phenotype, showing resistance to antibiotics from at least three different classes. Additionally, 94.8% of strains were resistant to at least seven antibiotics.

**Table 2:** Results of antimicrobial resistance of ESBL-producing *Enterobacteriaceae* from retail food in China.

**Antibiotics**	**R(%)**	**I(%)**	**S(%)**
AMP	94(97.9)	0(0)	2(2.1)
AMC	25(26.0)	44(45.8)	27(28.1)
AZM	49(51.0)	22(22.9)	25(26.0)
IPM	5(5.2)	0(0)	91(94.8)
MEM	3(3.1)	2(2.1)	91(94.8)
CN	50(52.1)	0(0)	46(47.9)
TM	57(59.4)	0(0)	39(40.6)
TE	80(83.3)	1(1.1)	15(15.6)
CIP	61(63.5)	0(0)	35(36.5)
LEV	58(60.4)	2(2.1)	36(37.5)
SXT	78(81.3)	0(0)	18(18.7)
SSS	89(92.7)	2(2.1)	5(5.2)
C	80(83.3)	2(2.1)	14(14.6)
KF	93(96.9)	0(0)	3(3.1)
FOX	6(6.3)	0(0)	90(93.7)
EFT	92(95.8)	0(0)	4(4.2)
CTX	94(97.8)	1(1.0)	1(1.0)
CAZ	41(42.7)	19(19.8)	36(37.5)
FEP	29(30.2)	17(17.7)	50(52.1)

*R, resistant; I, intermediate resistance; S, susceptibility*.

**Figure 1:**
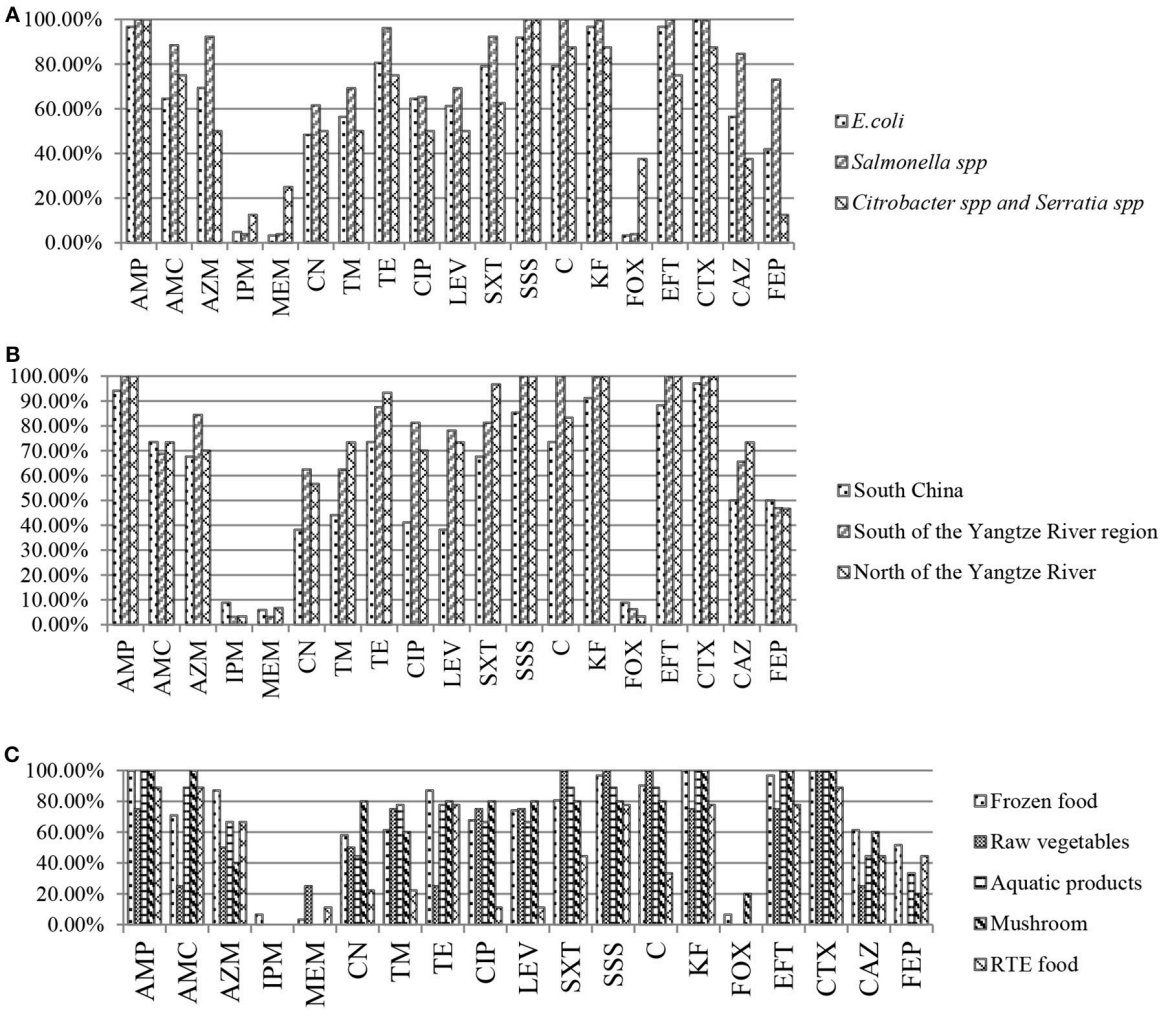
Results of antibiotic resistance rate of *Enterobacteriaceae* isolates from retail food in China. **(A)** Results of antibiotic resistance rate by species. **(B)** Results of antibiotic resistance rate by regions. **(C)** Results of antibiotic resistance rate by food types.

By species, more than 92% of *E. coli, Salmonella* spp., *Citrobacter* spp., and *Serratia* spp. strains showed resistance to AMP and SSS. High levels of resistance to CTX, KF, and EFT were observed for *E. coli* and *Salmonella* spp. strains (≥92%), *Citrobacter* spp. and *Serratia* spp. strains (75–87.5%). A high percentage of *E. coli, Salmonella* spp., *Citrobacter* spp., and *Serratia* spp. strains were resistant to antibiotics tested, such as TE, C, SXT, and AMC (≥60%) and LEV, CIP, TM and CN (48.4%-69.2%). Moreover, the highest prevalence of recorded was against AZM, CAZ, and FEP in *Salmonella* spp. strains (92.3, 84.6, and 73.1%), followed by *E. coli* (69.4, 56.5, and 41.9%), *Citrobacter* spp. and *Serratia* spp. (50, 37.5, and 12.5%). The resistance against carbapenems and FOX was low in *E. coli* and *Salmonella* spp. strains (3.2–4.8%), while 12.5, 25, and 37.5% of *Citrobacter* spp. and *Serratia* spp. strains showed resistance and intermediate resistance to IPM, MEM and FOX respectively (Figure 1A). By regions, high levels of resistance to AMP, SSS, EFT and CTX (≥85.3%), and AMC, AZM, TE, SXT and C (≥67.7%) were observed. Moreover, a high percentage of the isolates resistance to CIP and LEV (70–81.3%), TM and CN (56.7–73.4%) were observed in all regions except for South China. The resistance against carbapenems and FOX was low (3.1–8.8%) (Figure 1B). By food types, the highest prevalence recorded was against AMP, SSS, KF, EFT, and CTX (≥92%) in all kinds of retail food. A high percentage of the isolates were resistant to other antibiotics tested, such as AMC and TE (≥70%), CAZ (44.5–61.3%) in retail food except for raw vegetables. Moreover, high levels of resistance to CIP, LEV, SXT, and C (≥67.7%), TM (60–77.8%) and CN (44.5–80%) in retail food except for RTE foods were also observed. The resistance rate to FOX, carbapenems varied from 0 to 25% in retail food (Figure 1C). Overall, there were no statistically significant difference in the resistances to antimicrobials for ESBL-producing strains by species (Figure 1A), regions (Figure 1B), and food types (Figure 1C; *P* >0.05). In addition, 35 strains were isolated from 16 food samples which each one sample was detected 2–3 *E. coli* or *Salmonella* spp. strains, and the same species from the same food sample had the same resistant pattern. One isolate belong to each resistant pattern was selected from each food sample. Thus, 72 isolates which were selected from 96 ESBL-producing strains were analyzed for further study. Information on the 72 ESBL-producing strains is shown in Table 3.

**Table 3:** Results of β-lactamase gene types, integrons and transconjugants of β-lactamase -producing *Enterobacteriaceae* from retail food in China.

**No**.	**Strains**	**Species**	**Resources**	**City**	**β-lactamase phenotypes**	**Integrons**	**Transconjugants^a^**
1	121-0.1	*C. freundii*	Frozen chicken-meat	Guangzhou	CTX-M+TEM+CMY+OXA	Int1	+
2	1565-2	*E. coli*	Cucumber	Guangzhou	CTX-M+SHV	Int1	
3	1567-1	*E. coli*	Frozen pasta	Guangzhou	CTX-M+SHV	Int1	–
4	1573-3	*E. coli*	Chicken	Guangzhou	SHV+OXA	Int1	
5	1587-3	*E. coli*	Brine-soaked duck	Guangzhou	SHV	Int1	–
6	1650-1	*E. coli*	Mushroom	Guangzhou	CTX-M+SHV	Int1	
7	1501-3	*E. coli*	Pork	Guangzhou	TEM	Int1	–
8	1507-2	*E. coli*	Freshwater aquatic product	Guangzhou	TEM	Int1	
9	1511-3	*E. coli*	Brine-soaked chicken	Guangzhou	TEM	Int1	
10	1516-1	*E. coli*	Cold noodles in sauce	Guangzhou	CTX-M+TEM	Int1	+
11	1524-1	*E. coli*	Chicken	Guangzhou	SHV	Int1	–
12	1492-2	*E. coli*	Frozen chicken-meat	Guangzhou	CTX-M+TEM	Int1	
13	1845-2	*E. coli*	Frozen chicken-meat	Shaoguang	CTX-M	Int1	+
14	715-2	*C. koseri*	Frozen pasta	Fuzhou	TEM+OXA	Int1	–
15	2241-1	*E. coli*	Cucumber	Fuzhou	CTX-M+SHV	Int1	–
16	2245-1	*E. coli*	crozen sheep-meat	Fuzhou	CTX-M+SHV	Int1	–
17	55-19	*Salmonella* spp.	Chicken	Fuzhou	TEM	Int1	–
18	22-31	*C. amalonaticus*	Cold vegetable dish in sauce	Haikou	TEM+OXA	Int1	–
19	2403-2	*E. coli*	Pork	Haikou	CTX-M+TEM+OXA	Int1	+
20	2443-1	*E. coli*	Frozen chicken-meat	Haikou	CTX-M+TEM	Int1	–
21	2266-2	*E. coli*	Frozen pasta	Nanning	SHV+TEM+OXA	Int1	+
22	2293-2	*E. coli*	Frozen chicken-meat	Nanning	SHV+TEM+OXA	Int1	–
23	2295-1	*E. coli*	Mushroom	Nanning	TEM+OXA	Int1	–
24	58-8	*Salmonella* spp.	Duck	Beihai	CTX-M+TEM	Int1	–
25	1975-1	*E. coli*	Beef	Haerbing	CTX-M+SHV	Int1	–
26	2151-1	*C. freundii*	Pork	Lanzhou	CTX-M+SHV	Int1	–
27	2181-2	*C. freundii*	Coriander	Lanzhou	CTX-M+OXA	Int1	–
28	2103-2	*E. coli*	Pork	Taiyuan	CTX-M+SHV+TEM	Int1	+
29	2107-1	*E. coli*	Marine food product	Taiyuan	CTX-M+TEM	Int1	
30	2110-1	*E. coli*	Cold noodles in sauce	Taiyuan	CTX-M+SHV+TEM	Int1	+
31	2131-2	*E. coli*	Freshwater aquatic product	Taiyuan	CTX-M+TEM	Int1	
32	2104-2	*E. coli*	Marine food product	Taiyuan	CTX-M+SHV+TEM	Int1	+
33	2123-1	*E. coli*	Chicken	Taiyuan	CTX-M+TEM	Int1	–
34	53-19	*Salmonella* spp.	Duck	Taiyuan	CTX-M+TEM	Int1	+
35	53-21	*Salmonella* spp.	Frozen chicken-meat	Taiyuan	TEM	Int1	
36	53-25	*Salmonella* spp.	Frozen chicken-meat	Taiyuan	CTX-M+TEM	Int1	–
37	53-35	*Salmonella* spp.	Duck	Taiyuan	CTX-M+TEM	Int1	+
38	53-37	*Salmonella* spp.	Frozen chicken-meat	Taiyuan	CTX-M+TEM	Int1	–
39	2102-1	*E. coli*	Beef	Taiyuan	TEM+OXA	Int1	+
40	2111-2	*E. coli*	Cold noodles in sauce	Taiyuan	CTX-M+SHV+TEM	Int1	–
41	2069-1	*E. coli*	Mushroom	Beijing	CTX-M+TEM	Int1	–
42	2024-2	*C. freundii*	Marine food product	Jinan	CTX-M+TEM	–	+
43	2016-2	*E. coli*	Frozen pasta	Jinan	CTX-M+TEM	Int1	
44	2023-1	*E. coli*	Chicken	Jinan	SHV+TEM	Int1	+
45	2032-1	*E. coli*	Frozen chicken-meat	Jinan	SHV+TEM	Int1	–
46	2037-1	*E. coli*	Chicken	Jinan	CTX-M+SHV	Int1	+
47	2039-1	*E. coli*	Freshwater aquatic product	Jinan	CTX-M+TEM	Int1	–
48	2025-2	*E. coli*	Freshwater aquatic product	Jinan	CTX-M+SHV+TEM	Int1	–
49	2043-1	*E. coli*	Brine-soaked chicken	Jinan	CTX-M+SHV+TEM	Int1	–
50	30-14	*C. amalonaticus*	Freshwater aquatic product	Wuhan	CTX-M+TEM	Int1	–
51	2694-2	*E. coli*	Frozen chicken-meat	Wuhan	CTX-M+TEM	Int1	–
52	64-6	*Salmonella* spp.	Frozen chicken-meat	Wuhan	CTX-M+TEM	Int1	–
53	2699-2	*S. marcescens*	Mushroom	Wuhan	TEM	Int1	+
54	2684-2	*E. coli*	Roast duck	Wuhan	SHV+TEM	Int1	+
55	2679-2	*E. coli*	Freshwater aquatic product	Wuhan	CTX-M+TEM	Int1	–
56	2553-1	*E. coli*	Pork	Chengdou	CTX-M+TEM	Int1	–
57	2574-2	*E. coli*	Duck	Chengdou	CTX-M+TEM+OXA	Int1	+
58	2590-1	*E. coli*	Tomato	Chengdou	CTX-M+SHV+TEM	Int1	–
59	62-19	*Salmonella* spp.	Frozen chicken-meat	Chengdou	TEM+OXA	Int1	–
60	62-20	*Salmonella* spp.	Frozen pork	Chengdou	CTX-M+TEM	Int1	–
61	2603-2	*E. coli*	Pork	Hefei	CTX-M+SHV+TEM+OXA	Int1	+
62	2624-2	*E. coli*	Chicken	Hefei	SHV+TEM	Int1	–
63	2642-2	*E. coli*	Frozen chicken-meat	Hefei	TEM+OXA	Int1	+
64	63-13	*Salmonella* spp.	Frozen chicken-meat	Hefei	CTX-M+TEM+OXA	Int1	+
65	2644-3	*E. coli*	Frozen chicken-meat	Hefei	CTX-M+TEM	–	–
66	61-1	*Salmonella* spp.	Chicken	Nanchang	CTX-M+TEM	Int1	+
67	2523-2	*E. coli*	Chicken	Nanchang	TEM	Int1	
68	2543-1	*E. coli*	Frozen chicken-meat	Nanchang	CTX-M+TEM	Int1	–
69	2722-2	*E. coli*	Pork	Shanghai	CTX-M+SHV+TEM	Int1	–
70	2723-1	*E. coli*	Chicken	Shanghai	SHV+TEM	Int1	
71	2745-2	*E. coli*	Frozen chicken-meat	Shanghai	CTX-M+SHV+TEM	Int1	+
72	2749-2	*E. coli*	Mushroon	Shanghai	CTX-M+TEM	Int1	–

a *+, have been obtained transconjugants; –, have not been obtained transconjugants*.

### Characterization of β-lactamase genes

One or more β-lactamase genes were detected in all of the 72 isolated strains. The TEM type was clearly prevalent in our isolates (81.9%), followed by the CTX-M type (68.1%), SHV type (38.9%), and OXA type (20.8%). CTX-M in combination with TEM β-lactamase (34.7%) was the most frequent ESBL type. TEM β-lactamase was detected alone in seven isolates (four *E. coli*, one *Serratia marcescens*, and two *Salmonella* spp.). SHV ESBL was only detected in *E. coli* isolates (alone or in combination with other types of β-lactamases), but for one *C. freundii* 2151-1 isolate, was identified in combination with CTX-M. OXA β-lactamase was detected in combination with other types of β-lactamases (Table 3).

### Prevalence of ESBL-producing *enterobacteriaceae* in retail food products

**Figure 2:**
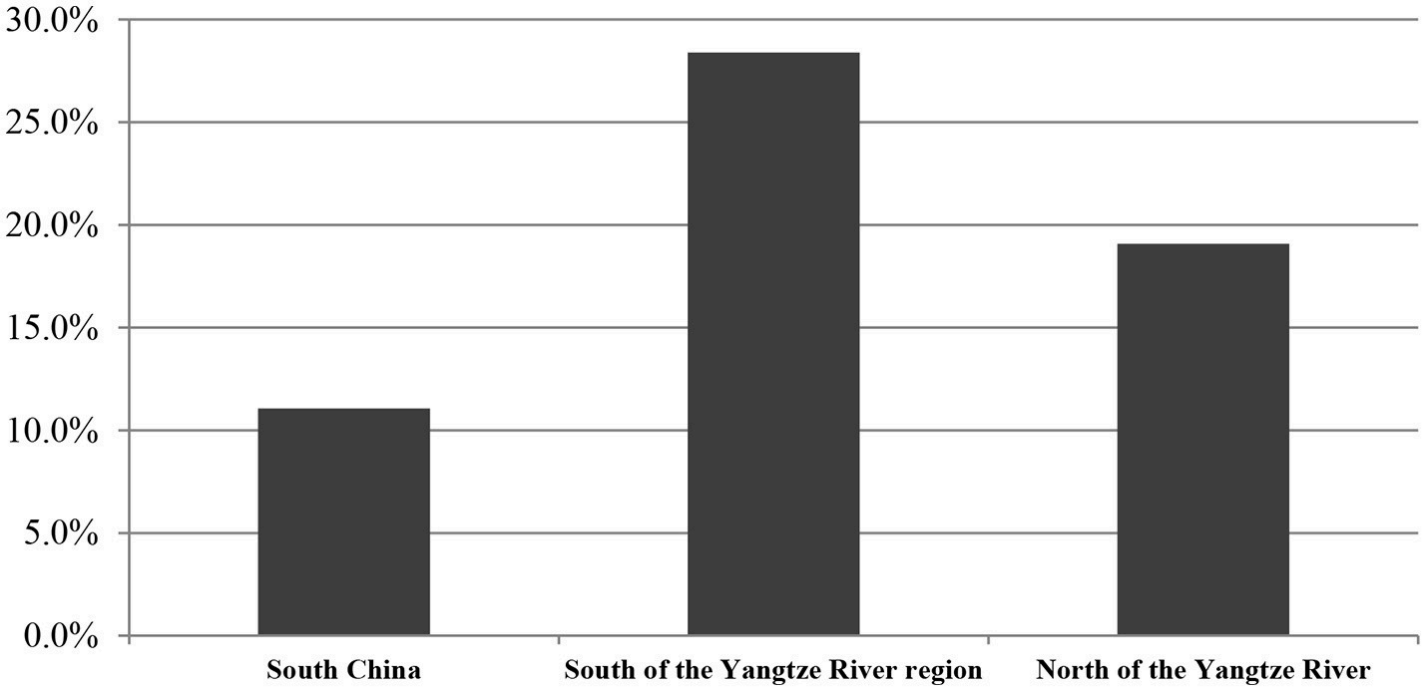
The occurrence of ESBL- producing *Enterobacteriaceae* isolated from retail foods by regions in China. % of isolates were include the prevalence of resistance or intermediate resistance to the antibiotics. AMP, ampicillin; AMC, amoxicillin/clavulanic acid; AZM, aztreonam; IPM, imipinem; MEM, meropenem; CN, gentamicin; TM, tobramycin; TE, tetracycline; CIP, ciprofloxacin; LEV, levofloxacin; SXT, trimethoprim/sulfamethoxazole; SSS, sulfonamide; C, chloramphenicol; KF, cephalothin; FOX, cefoxitin; EFT, ceftiofur; CTX, cefotaxime; CAZ, ceftazidime; FEP, cefepime.

From our analysis of 429 food samples using the disk diffusion method, we detected ESBL-producing *Enterobacteriaceae* in 72 (16.8%). Some specific differences were present among distinct food varieties. For example, ESBL-positive *Enterobacteriaceae* were detected in 23 of 70 (32.9%) frozen food samples, five of 29 (27.2%) mushroom samples, 23 of 131 (17.6%) raw meats, four of 30 (13.3%) fresh vegetables, eight of 72 (11.1%) RTE foods, and nine of 97 (9.3%) aquatic products (Table 4). By regions, our study detected ESBL-positive *Enterobacteriaceae* in 16 of 24 cities surveyed (66.7%), with prevalence ranging from 6.7% in cities in ha'erbin to 72.7% in Jinan. The prevalence of ESBL-positive *Enterobacteriaceae* varied with different latitude: 24 of 217 foods collected in South China (11.1%), 25 of 131 foods collected in North of the Yangtze River region (19.1%), and 23 of 81 foods collected in South of the Yangtze River region (28.4%) were positive for ESBL-positive *Enterobacteriaceae* (Figure 2). Overall, there were no statistically significant difference in the prevalence of ESBL-positive *Enterobacteriaceae* by regions (Figure 2) and by food type (Table 4; *P* > 0.05).

**Table 4:** The occurrence of ESBL- producing *Enterobacteriaceae* isolated from retail foods in China.

**Product category**	**Total tests**	**ESBL isolates (No.)**	**ESBL isolates (%)**
Raw meat	131	23	17.6
Chicken	42	10	23.8
Duck	23	4	17.4
Pork	51	7	13.7
Beef	15	2	13.3
Aquatic products	97	9	9.3
Marine food product	40	3	7.5
Freshwater aquatic product	57	6	10.5
Frozen food	70	23	32.9
Frozen chicken-meat	40	17	42.5
Frozen pork	3	1	33.3
Frozen pasta	19	4	21.1
Frozen sheep-meat	7	1	14.3
Frozen beef	1	0	0
Raw vegetables	30	4	13.3
Cucumber	8	2	25.0
Tomato	5	1	20.0
Coriander	6	1	16.7
Lettuce	11	0	0
RTE food	72	8	11.1
Cold noodles in sauce	11	3	27.3
Brine-soaked chicken	10	2	20.0
Cold vegetable dish in sauce	7	1	14.3
Brine-soaked duck	8	1	12.5
Roast duck	12	1	8.3
Brine-soaked pork	14	0	0
Roast pork	4	0	0
Roast chicken	3	0	0
Brine-soaked beef	2	0	0
Milk	1	0	0
Mushroom	29	5	17.2
Total	429	72	16.8

### Detection of integrons

Class 1 integrons were present in 70 of the 72 ESBL strains, whereas class 2 integrons were not identified in this collection (Table 3).

### Conjugal transfer of ESBL-encoding genes

In the conjugation experiments, transfer of the ESBL phenotype was demonstrated in 22 out of 72 ESBL strains tested (Table 3); however, transconjugants could not be recovered in the other strains. All of the obtained transconjugants received β-lactamase genes and acquired the ESBL phenotype. In addition, seven transconjugants acquired the ESBL phenotype and resistance to SSS, CIP, CN, SXT, TE, and C (Table 5).

**Table 5:** Characterization of transconjugants of β-lactamase -producing *Enterobacteriaceae* from retail foods in China.

**Strains**	**Species**	**β-lactamase phenotypes**	**Antibiotic profiles**
121-0.1	*C. freundii*	CTX-M+TEM+CMY+OXA	AMP-KF-EFT-FOX-CTX
1516-1	*E. coli*	CTX-M+TEM	AMP-AZM-KF-EFT-CTX
1845-2	*E. coli*	CTX-M	AMP-AMC-KF-EFT-CTX-SSS
2403-2	*E. coli*	CTX-M+TEM+OXA	AMP-AZM-FEP-KF-EFT-CTX-CAZ
2266-2	*E. coli*	SHV+TEM+OXA	AMP-KF-EFT-CTX
2103-2	*E. coli*	CTX-M+SHV+TEM	AMP-KF-EFT-CTX-CIP
2110-1	*E. coli*	CTX-M+SHV+TEM	AMP-KF-EFT-CTX
2104-2	*E. coli*	CTX-M+SHV+TEM	AMP-KF-EFT-CTX
53-19	*Salmonella* spp.	CTX-M+TEM	AMP-AZM-KF-EFT-CTX-CAZ-CN
53-35	*Salmonella* spp.	CTX-M+TEM	AMP-AZM-FEP-KF-EFT-FOX-CTX-CAZ
2102-1	*E. coli*	TEM+OXA	AMP-KF-EFT-FOX-CTX-CAZ
2024-2	*C. freundii*	CTX-M+TEM	AMP-AZM-KF-EFT-CTX
2023-1	*E. coli*	SHV+TEM	AMP-KF-EFT-CTX-CAZ
2037-1	*E. coli*	CTX-M+SHV	AMP-KF-EFT-CTX-CAZ-CIP
2699-2	*S. marcescens*	TEM	AMP-AZM-KF-EFT-FOX-CTX-CAZ
2684-2	*E. coli*	SHV+TEM	AMP-AZM-KF-EFT-CTX-SXT
2574-2	*E. coli*	CTX-M+TEM+OXA	AMP-KF-EFT-FOX-CTX-CAZ
2603-2	*E. coli*	CTX-M+SHV+TEM+OXA	AMP-AZM-KF-EFT-CTX
2642-2	*E. coli*	TEM+OXA	AMP-FEP-KF-EFT-CTX-CAZ
63-13	*Salmonella* spp.	CTX-M+TEM+OXA	AMP-KF-EFT-CTX-C
61-1	*Salmonella* spp.	CTX-M+TEM	AMP-KF-EFT-CTX-TE
2745-2	*E. coli*	CTX-M+SHV+TEM	AMP-KF-EFT-CTX

## Discussion

Contaminated food is a major source of gastrointestinal microbial pathogens and has caused numerous foodborne disease outbreaks in the developing world. Because of the wide use of third- and fourth-generation cephalosporin antibiotics, the prevalence of multidrug-resistant *Enterobacteriaceae* has increased yearly, around the globe. The production of ESBLs is the primary mechanism of antibiotic resistance in *Enterobacteriaceae*, and ESBL-producing isolates are widespread in China and other countries. Accordingly, antibiotic resistance owing to ESBLs is a major public health concern. Although ESBL-producing isolates have not been frequently reported in food products, some reported have described ESBL-producing isolates in chicken, pork, beef, other raw meats (Ojer-Usoz et al., 2013; Tekiner and Özpinar, 2016), and even fresh vegetables (Reuland et al., 2014; Ben Said et al., 2015; Kim et al., 2015; Nguyen et al., 2016; Freitaga et al., 2018). Additionally, some recent studies have been undertaken to determine the prevalence of ESBL-producing *Enterobacteriaceae* from animals or animal sources of foods in China (Zheng et al., 2012; Yang et al., 2014) and fewer reports have described other types of foods (Yang et al., 2014). Moreover, in these studies, only a limited number of food samples or food sources of a particular type were examined, and no systematic studies have been conducted on a nationwide scale in China.

This is the first nationwide survey of the prevalence of ESBL-producing *Enterobacteriaceae* in foods in China. This survey was a large-scale investigation of ESBL-producing *Enterobacteriaceae* in fresh, frozen, and RTE food products collected from more of the provincial capitals of China, and the types of food sampled were more culturally relevant than those in some previous studies. Of the 429 food samples examined, we found an overall prevalence of 16.8%, with certain differences being present among the distinct food varieties. Moreover, ESBL-producing *Enterobacteriaceae* varied from 9.3% of 97 isolates in aquatic products to 32.9% of 70 isolates in frozen food samples. This result was higher than those found by van Hoek et al. (2015) (3.5% of 1216 isolates), Reuland et al. (2014) (6% of 119 isolates), and Maravić et al. (2015) (4.2% of 1351 isolates). Our results indicated ESBL-producing *Enterobacteriaceae* has the highest incidence in frozen foods (32.9% of 70 isolates), ranging from 14.3% of 7 isolates in frozen sheep-meat to 42.5% of 40 isolates in frozen chicken-meat. The risks of ESBL-producing strains in these food products should draw public attention. Raw meat products have often been suggested as the main source of ESBL-producing *Enterobacteriaceae*, and several studies were carried out to assess the prevalence of ESBL-producing *Enterobacteriaceae* in these products. 23 of the 131 raw meat samples were confirmed positive for the presence of ESBL-producing Enterobacteriaceae (17.6%), which is comparable to results from some other countries (Schilla et al., 2017) (20.6% of 63 isolates). Prevalence in poultry products (23.8% of 42 isolates in chicken and 17.4% of 23 isolates in duck) was higher than in pork (13.7% of 51 isolates) or beef (13.3% of 15 isolates), which is a lower prevalence than has been reported for many countries (84% of 45 isolates in poultry, 55% of 47 isolates in pork and 59% of 49 isolates in beef) (Ojer-Usoz et al., 2013). The prevalence of ESBL-producing *Enterobacteriaceae* in frozen foods was higher than that in raw meat products, indicating that frozen foods may be susceptible to cross-contamination in the production process. Our results found a high prevalence of ESBL-producing *Enterobacteriaceae* in raw vegetables (13.3% of 30 isolates) than previous studies (3.5% of 1216 isolates) (van Hoek et al., 2015). RTE foods such as cold noodles in sauce, cold vegetable dish in sauce, brine-soaked or roast meat (chicken, duck, pork and beef) and milk are commonly consumed in China. Of the 72 RTE food samples examined, we found an overall prevalence of 11.1%, with some differences among different varieties of food. No ESBL-producing *Enterobacteriaceae* were isolated from brine-soaked or roast pork, brine-soaked beef, milk and roast chicken. By contrast, a high prevalence of ESBL-producing *Enterobacteriaceae* in cold noodles in sauce (27.3% of 11 isolates), brine-soaked chicken (20% of 10 isolates), cold vegetable dish in sauce (14.3% of 7 isolates), brine-soaked duck (12.5% of 8 isolates) and roast duck (8.3% of 12 isolates) was observed, and we still need to pay attention to contamination of these food.

Categorized by genera, *E. coli* was the predominant ESBL producer, consistent with the results of other studies (Lu et al., 2010; Mokracka et al., 2012; Korzeniewska and Harnisz, 2013). However, the rate of ESBL-positive *E. coli* was lower than those found in clinical settings (Xiao et al., 2015) (42.3% of 634 isolates), but is consistent with a previous report in animals in China (Zheng et al., 2012) (13.1% of 896 isolates). *Citrobacter* and *Serratia* are mainly detected in the clinical setting and were present at lower prevalence in our study than in previous clinical reports (Parka et al., 2005). The differences in the occurrence of ESBL-producing *Enterobacteriaceae* in retail foods may also be associated with regional differences. Our study detected ESBL-positive *Enterobacteriaceae* in 16 of 24 cities surveyed (66.7%), with prevalence ranging from 6.7% in cities in ha'erbin to 72.7% in Jinan. The prevalence of ESBL-positive *Enterobacteriaceae* varied with different latitude, and the most occurrence was observed in South of the Yangtze River region (28.4%), followed in North of the Yangtze River region (19.1%) and in South China (11.1%). Thus, measures for governmental agencies to play a larger role in controlling the risks of ESBL-producing *Enterobacteriaceae* are recommended.

Many studies have shown that the generation of ESBL enzymes is the primary mechanism of antibiotic resistance in *Enterobacteriaceae*. ESBL-producing *Enterobacteriaceae* can exhibit strong antimicrobial resistance to penicillins, third-generation cephalosporins, aminoglycosides, C, quinolones, and TE (Rupp and Fey, 2003). In this study, we found that over 50% of ESBL-positive strains were resistant to AZM, TE, SXT, SSS, C, KF, CTX, and EFT, and these results are consistent with a previous report (Yang et al., 2014). Currently, the most reliable therapies used for infection by ESBL-producing *Enterobacteriaceae* are FEP and carbapenems (Paterson et al., 2001). In the present study, 47.9% of β-lactamase-producing strains showed resistance to FEP, in contrast with previous studies (Chen et al., 2012; Maravić et al., 2015). This phenomenon may be explained by the varied use of FEP in different countries. Previous studies have reported that few ESBL-positive isolates were resistant to carbapenems, even in the clinical setting (Chen et al., 2014; Hu et al., 2015). However, 5.2% of strains were resistant to IPM and MEM in the present study. Nevertheless, carbapenems may still be effective for the treatment of ESBL-producing *Enterobacteria*. Taken together, these data demonstrated that the emergence of ESBL-producing strains and the wide use of β-lactam antimicrobial agents had a direct causal relationship; therefore, measures for controlling the overuse of third-generation cephalosporins should be implemented to reduce the emergence of drug-resistant strains.

ESBLs include TEM-, SHV-, OXA-, and CTX-M-type enzymes (Livermore, 2008; Pfeiier et al., 2010). In the 1980s, SHV- and TEM-type ESBLs were the primary cause of third-generation cephalosporin resistance among *Enterobacteriaceae* (Pitout, 2012). In contrast, since 2000, the CTX-M-type ESBL became predominant over TEM- and SHV-type enzymes (Cantón et al., 2012; Peirano et al., 2012; Doi et al., 2013). Because different countries and regions have different strategies for antibiotics use, the dominance of ESBL-enzyme types differs among countries. CTX-M has been reported to be dominant in China and is the most common type of ESBL globally (Cantón et al., 2012; Peirano et al., 2012). Genotypic characterization by PCR assays revealed that blaTEM was the most frequent gene, followed by blaCTX-M and blaSHV, consistent with previous reports in other countries (Ferreira et al., 2014; Kawamura et al., 2014; Zarfel et al., 2014). Class 1 integrons were present in 70 of the 72 ESBL -producing strains, but class 2 integrons were not identified in this collection, which was consistent with other reports (Schmiedel et al., 2014; Ben Said et al., 2015). In the conjugation transfer experiments, 22 isolates were detected as transconjugants, demonstrating the prevalence of ESBL-producing *Enterobacteriaceae* in China. Moreover, transconjugants received the β-lactamase gene and showed resistance to β-lactam antibiotics and some non-β-lactam antibiotics. These results indicated that plasmids could not only carry genes encoding ESBL β-lactamases but also could spread horizontally among *Enterobacteriaceae*, thereby contributing to resistance to antimicrobials, including β-lactams and some non-β-lactams, resulting in high prevalence of multidrug resistance.

We here report the results of the first investigation of the prevalence of ESBL- -producing *Enterobacteriaceae* isolated from retail foods in China. Our results showed that frozen foods, mushrooms, raw meat products, raw vegetables, and aquatic products were important vehicles for the dissemination of ESBL-producing *Enterobacteria* and posed a critical health risk for Chinese consumers. Therefore, close surveillance of antimicrobial resistance in bacteria from food-producing and food-derived products should be established as a priority. In addition, our results could provide a basis for further investigations into ESBL-positive isolates from food products in order to avoid jeopardizing the safety of final products.

## Author contributions

QY contributed to designed the work that led to the submission, acquired data, and played an important role in interpreting the results. QW contributed to revised the manuscript and approved the final version. SZ, GY helped to acquired data. JZ, MC, LX, and JW helped perform the analysis with constructive discussions.

### Conflict of interest statement

The authors declare that the research was conducted in the absence of any commercial or financial relationships that could be construed as a potential conflict of interest.
