# Supplementary material for: Characterization of Extended-Spectrum β-Lactamase-Producing Enterobacteriaceae From Retail Food in China
Source: Front Microbiol. 2018 Aug 8;9:1709. doi: 10.3389/fmicb.2018.01709 (PMC6092486; doi:10.3389/fmicb.2018.01709)
Supplement: Supplementary file 1 [file Table_1.docx]

**Supplementary Material**

Table S1 Information of 429 food Samples used in paper.

| No. | Resources | Sample No. | City | No. | Resources | Sample No. | City | No. | Resources | Sample No. | City |
| --- | --- | --- | --- | --- | --- | --- | --- | --- | --- | --- | --- |
| 1 | freshwater aquatic product | YXJ1428 | Guangzhou | 40 | freshwater aquatic product | JNJ2039 | Jinan | 79 | freshwater aquatic product | HFJ2627 | Hefei |
| 2 | freshwater aquatic product | LWC1456 | Guangzhou | 41 | freshwater aquatic product | JNJ2024 | Jinan | 80 | marine food product | HFC2606 | Hefei |
| 3 | marine food product | LWC1457 | Guangzhou | 42 | freshwater aquatic product | BJC2055 | Beijing | 81 | freshwater aquatic product | WHJ2679 | Wuhan |
| 4 | freshwater aquatic product | LWN1477 | Guangzhou | 43 | freshwater aquatic product | BJC2056 | Beijing | 82 | freshwater aquatic product | WHC2655 | Wuhan |
| 5 | freshwater aquatic product | LWN1478 | Guangzhou | 44 | marine food product | BJJ2077 | Beijing | 83 | freshwater aquatic product | WHC2657 | Wuhan |
| 6 | freshwater aquatic product | LWN1480 | Guangzhou | 45 | freshwater aquatic product | BJJ2080 | Beijing | 84 | freshwater aquatic product | WHJ2678 | Wuhan |
| 7 | freshwater aquatic product | LWN1481 | Guangzhou | 46 | marine food product | TYC2104 | Taiyuan | 85 | marine food product | SHJ2727 | Shaihai |
| 8 | freshwater aquatic product | PYC1505 | Guangzhou | 47 | marine food product | TYC2107 | Taiyuan | 86 | freshwater aquatic product | SHJ2729 | Shaihai |
| 9 | freshwater aquatic product | PYC1506 | Guangzhou | 48 | marine food product | TYJ2129 | Taiyuan | 87 | marine food product | YXC024 | Guangzhou |
| 10 | freshwater aquatic product | PYC1507 | Guangzhou | 49 | freshwater aquatic product | TYJ2131 | Taiyuan | 88 | freshwater aquatic product | FCJ084 | Fuzhou |
| 11 | freshwater aquatic product | PYN1528 | Guangzhou | 50 | freshwater aquatic product | LZC2155 | Lanzhou | 89 | marine food product | FCJ079 | Fuzhou |
| 12 | freshwater aquatic product | PYN1529 | Guangzhou | 51 | freshwater aquatic product | LZC2156 | Lanzhou | 90 | marine food product | ZCJ223 | Guangzhou |
| 13 | freshwater aquatic product | PYN1531 | Guangzhou | 52 | marine food product | LZJ2175 | Lanzhou | 91 | marine food product | SZC254 | Shenzhen |
| 14 | freshwater aquatic product | CHC1556 | Guangzhou | 53 | freshwater aquatic product | LZJ2189 | Lanzhou | 92 | freshwater aquatic product | SGN339 | Shaoguang |
| 15 | freshwater aquatic product | CHC1557 | Guangzhou | 54 | marine food product | LZJ2191 | Lanzhou | 93 | freshwater aquatic product | HKC507 | Haikou |
| 16 | freshwater aquatic product | CHJ1577 | Guangzhou | 55 | freshwater aquatic product | FZC2203 | Fuzhou | 94 | freshwater aquatic product | SYC556 | Shengyang |
| 17 | marine food product | CHJ1578 | Guangzhou | 56 | marine food product | FZC2205 | Fuzhou | 95 | freshwater aquatic product | SYN589 | Shengyang |
| 18 | freshwater aquatic product | CHJ1579 | Guangzhou | 57 | marine food product | NNC2254 | Nanchang | 96 | freshwater aquatic product | SYN590 | Shengyang |
| 19 | marine food product | ZCC1604 | Guangzhou | 58 | marine food product | FZC2206 | Fuzhou | 97 | freshwater aquatic product | WHJ976 | Wuhan |
| 20 | marine food product | ZCC1605 | Guangzhou | 59 | marine food product | FZC2207 | Fuzhou | 98 | pork | LWC1451 | Guangzhou |
| 21 | marine food product | ZCC1606 | Guangzhou | 60 | marine food product | FZJ2226 | Fuzhou | 99 | pork | LWC1452 | Guangzhou |
| 22 | freshwater aquatic product | ZCJ1629 | Guangzhou | 61 | marine food product | FZJ2228 | Fuzhou | 100 | pork | LWC1453 | Guangzhou |
| 23 | freshwater aquatic product | ZCJ1630 | Guangzhou | 62 | marine food product | NNJ2281 | Nanning | 101 | pork | LWN1472 | Guangzhou |
| 24 | freshwater aquatic product | ZCJ1631 | Guangzhou | 63 | marine food product | BHJ2377 | Beihai | 102 | pork | LWN1475 | Guangzhou |
| 25 | marine food product | SZC1654 | Shenzhen | 64 | freshwater aquatic product | HKC2406 | Haikou | 103 | pork | PYC1501 | Guangzhou |
| 26 | marine food product | SZC1657 | Shenzhen | 65 | freshwater aquatic product | HKJ2427 | Haikou | 104 | pork | PYC1502 | Guangzhou |
| 27 | freshwater aquatic product | SZJ1679 | Shenzhen | 66 | marine food product | HKJ2428 | Haikou | 105 | pork | PYC1503 | Guangzhou |
| 28 | freshwater aquatic product | SZJ1681 | Shenzhen | 67 | freshwater aquatic product | HKJ2429 | Haikou | 106 | duck | LWN1473 | Guangzhou |
| 29 | freshwater aquatic product | ZJJ1778 | Zhanjiang | 68 | marine food product | SYC2454 | Shengyang | 107 | duck | LWN1474 | Guangzhou |
| 30 | freshwater aquatic product | ZJJ1780 | Zhanjiang | 69 | marine food product | SYC2455 | Shengyang | 108 | pork | PYN1522 | Guangzhou |
| 31 | freshwater aquatic product | SGC1805 | Shaoguang | 70 | marine food product | SYC2456 | Shengyang | 109 | pork | PYN1525 | Guangzhou |
| 32 | freshwater aquatic product | SGJ1828 | Shaoguang | 71 | marine food product | SYC2457 | Shengyang | 110 | duck | PYN1523 | Guangzhou |
| 33 | marine food product | SGJ1829 | Shaoguang | 72 | marine food product | SYJ2476 | Shengyang | 111 | chicken | PYN1524 | Guangzhou |
| 34 | freshwater aquatic product | SGJ1830 | Shaoguang | 73 | marine food product | SYJ2478 | Shengyang | 112 | pork | PYN1535 | Guangzhou |
| 35 | marine food product | HEBC1955 | Haerbin | 74 | freshwater aquatic product | SYJ2480 | Shengyang | 113 | pork | PYN1537 | Guangzhou |
| 36 | marine food product | HEBC1956 | Haerbin | 75 | freshwater aquatic product | SYJ2481 | Shengyang | 114 | pork | CHC1551 | Guangzhou |
| 37 | marine food product | HYJ1877 | Heyuan | 76 | marine food product | NCJ2523 | Nanchang | 115 | chicken | CHC1552 | Guangzhou |
| 38 | freshwater aquatic product | HEBJ1978 | Haerbin | 77 | marine food product | NCJ2524 | Nanchang | 116 | pork | CHC1553 | Guangzhou |
| 39 | freshwater aquatic product | JNJ2025 | Jinan | 78 | freshwater aquatic product | CDJ2578 | Chengdou | 117 | pork | CHJ1572 | Guangzhou |
| 118 | chicken | CHJ1573 | Guangzhou | 160 | chicken | LZ213 | Lanzhou | 202 | chicken | NCJ2529 | Nanchang |
| 119 | duck | CHJ1574 | Guangzhou | 161 | beef | FZC2201 | Fuzhou | 203 | beef | NCJ2530 | Nanchang |
| 120 | pork | ZCC1601 | Guangzhou | 162 | pork | FZC2202 | Fuzhou | 204 | beef | NCJ2543 | Nanchang |
| 121 | pork | ZCC1603 | Guangzhou | 163 | pork | FZJ2222 | Fuzhou | 205 | chicken | NC294 | Nanchang |
| 122 | pork | ZCJ1622 | Guangzhou | 164 | chicken | FZJ2223 | Fuzhou | 206 | pork | CDC2551 | Chengdou |
| 123 | chicken | ZCJ1623 | Guangzhou | 165 | duck | FZJ2224 | Fuzhou | 207 | beef | CDC2552 | Chengdou |
| 124 | duck | ZCJ1624 | Guangzhou | 166 | chicken | FZ251 | Fuzhou | 208 | pork | CDC2553 | Chengdou |
| 125 | chicken | SZJ1673 | Shenzhen | 167 | chicken | FZ252 | Fuzhou | 209 | chicken | CDJ2573 | Chengdou |
| 126 | chicken | SZ141 | Shenzhen | 168 | chicken | FZ255 | Fuzhou | 210 | duck | CDJ2574 | Chengdou |
| 127 | beef | ZJC1752 | Zhanjiang | 169 | pork | NNC2253 | Nanning | 211 | chicken | CD301 | Chengdou |
| 128 | pork | ZJC1753 | Zhanjiang | 170 | duck | NN258 | Nanning | 212 | duck | CD302 | Chengdou |
| 129 | pork | ZJJ1772 | Zhanjiang | 171 | chicken | NN259 | Nanning | 213 | duck | CD303 | Chengdou |
| 130 | duck | ZJJ1774 | Zhanjiang | 172 | chicken | NN262 | Nanning | 214 | pork | HFC2603 | Hefei |
| 131 | chicken | HYC1853 | Heyuan | 173 | beef | XMC2302 | Xiamen | 215 | pork | HFJ2622 | Hefei |
| 132 | pork | HYJ1872 | Heyuan | 174 | chicken | XMJ2323 | Xiamen | 216 | chicken | HFJ2624 | Hefei |
| 133 | pork | XAC1901 | Xi’an | 175 | pork | BHC2353 | Beihai | 217 | chicken | WH315 | Wuhan |
| 134 | pork | XAC1903 | Xi’an | 176 | duck | BHJ2374 | Beihai | 218 | chicken | WH318 | Wuhan |
| 135 | pork | HEBC1951 | Haerbin | 177 | duck | XM265 | Xiamen | 219 | pork | SHJ2722 | Shaihai |
| 136 | pork | HEBC1953 | Haerbin | 178 | chicken | XM269 | Xiamen | 220 | chicken | SHJ2723 | Shaihai |
| 137 | chicken | HYJ1873 | Heyuan | 179 | chicken | BH272 | Beihai | 221 | duck | SHJ2724 | Shaihai |
| 138 | pork | HEBJ1972 | Haerbin | 180 | duck | BH274 | Beihai | 222 | beef | SHJ2725 | Shaihai |
| 139 | duck | HEBJ1974 | Haerbin | 181 | pork | HKC2403 | Haikou | 223 | chicken | FCJ083 | Fuzhou |
| 140 | beef | HEBJ1975 | Haerbin | 182 | beef | HKC2402 | Haikou | 224 | beef | SZJ271 | Shenzhen |
| 141 | duck | HYJ1874 | Heyuan | 183 | pork | HKJ2422 | Haikou | 225 | duck | SZJ272 | Shenzhen |
| 142 | chicken | JNJ2023 | Jinan | 184 | chicken | HKJ2423 | Haikou | 226 | chicken | SZN286 | Shenzhen |
| 143 | chicken | JNJ2037 | Jinan | 185 | duck | HKJ2424 | Haikou | 227 | beef | ZJJ371 | Zhanjiang |
| 144 | pork | BJJ2072 | Beijing | 186 | chicken | HK280 | Haikou | 228 | duck | SYN 586 | Shengyang |
| 145 | pork | TYC2101 | Taiyuan | 187 | duck | HK282 | Haikou | 229 | lettuce | LWC1464 | Guangzhou |
| 146 | beef | TYC2102 | Taiyuan | 188 | chicken | HK283 | Haikou | 230 | lettuce | LWN1491 | Guangzhou |
| 147 | pork | TYC2103 | Taiyuan | 189 | pork | SYC2451 | Shengyang | 231 | lettuce | PYN1541 | Guangzhou |
| 148 | beef | BJJ2074 | Beijing | 190 | beef | SYC2452 | Shengyang | 232 | lettuce | CHC1563 | Guangzhou |
| 149 | pork | TYJ2122 | Taiyuan | 191 | pork | SYC2453 | Shengyang | 233 | coriander | CHC1564 | Guangzhou |
| 150 | chicken | TYJ2123 | Taiyuan | 192 | pork | SYJ2472 | Shengyang | 234 | cucumber | CHC1565 | Guangzhou |
| 151 | duck | TYJ2124 | Taiyuan | 193 | chicken | SYJ2473 | Shengyang | 235 | lettuce | STC1713 | Shantou |
| 152 | chicken | TY203 | Taiyuan | 194 | duck | SYJ2474 | Shengyang | 236 | lettuce | ZJJ1791 | Zhanjiang |
| 153 | pork | LZC2152 | Lanzhou | 195 | chicken | SY286 | Shengyang | 237 | lettuce | HYC1863 | Heyuan |
| 154 | pork | LZC2151 | Lanzhou | 196 | chicken | SY290 | Shengyang | 238 | cucumber | BJC2065 | Beijing |
| 155 | pork | LZC2153 | Lanzhou | 197 | pork | NCC2501 | Nanchang | 239 | coriander | JNJ2031 | Jinan |
| 156 | beef | LZJ2172 | Lanzhou | 198 | pork | NCC2503 | Nanchang | 240 | cucumber | LZC2164 | Lanzhou |
| 157 | chicken | LZJ2187 | Lanzhou | 199 | pork | NCC2504 | Nanchang | 241 | coriander | LZJ2181 | Lanzhou |
| 158 | chicken | LZJ2173 | Lanzhou | 200 | pork | NCJ2522 | Nanchang | 242 | tomato | FZC2213 | Fuzhou |
| 159 | chicken | LZJ2198 | Lanzhou | 201 | chicken | NCJ2528 | Nanchang | 243 | lettuce | FZC2214 | Fuzhou |
| 244 | tomato | FZJ2240 | Fuzhou | 288 | cold noodles in sauce | LZC2160 | Lanzhou | 332 | frozen pasta | JNJ2048 | Jinan |
| 245 | cucumber | FZJ2241 | Fuzhou | 289 | brine-soaked chicken | LZC2161 | Lanzhou | 333 | frozen pasta | JNC2016 | Jinan |
| 246 | coriander | SYC2464 | Shengyang | 290 | brine-soaked chicken | TYJ2138 | Taiyuan | 334 | frozen pasta | BJC2066 | Beijing |
| 247 | lettuce | SYJ2490 | Shengyang | 291 | brine-soaked chicken | TYJ2139 | Taiyuan | 335 | frozen pasta | CHC1566 | Guangzhou |
| 248 | cucumber | SYJ2491 | Shengyang | 292 | roast pork | LZJ2179 | Lanzhou | 336 | frozen pasta | CHC1567 | Guangzhou |
| 249 | tomato | CDC2563 | Chengdou | 293 | brine-soaked pork | LZJ2180 | Lanzhou | 337 | frozen pasta | ZCC1618 | Guangzhou |
| 250 | tomato | CDJ2590 | Chengdou | 294 | cold vegetable dish in sauce | FZC2209 | Fuzhou | 338 | frozen pasta | HYC1867 | Heyuan |
| 251 | cucumber | CDJ2591 | Chengdou | 295 | brine-soaked duck | FZC2211 | Fuzhou | 339 | frozen pasta | HYC1868 | Heyuan |
| 252 | tomato | WHJ2690 | Wuhan | 296 | roast duck | FZJ2234 | Fuzhou | 340 | frozen pasta | LZC2167 | Lanzhou |
| 253 | cucumber | WHC2665 | Wuhan | 297 | brine-soaked chicken | FZJ2236 | Fuzhou | 341 | frozen pasta | NNC2266 | Nanchang |
| 254 | lettuce | SHC2713 | Shaihai | 298 | roast pork | LZJ2188 | Lanzhou | 342 | frozen pasta | HKC2416 | Haikou |
| 255 | cucumber | SHJ2741 | Shaihai | 299 | roast chicken | LZJ2194 | Lanzhou | 343 | frozen pasta | CDC2566 | Chengdou |
| 256 | coriander | CHJ181 | Guangzhou | 300 | brine-soaked duck | NNC2261 | Nanchang | 344 | frozen pasta | HKC2417 | Haikou |
| 257 | lettuce | SYC563 | Shengyang | 301 | brine-soaked pork | HKC2410 | Haikou | 345 | frozen chicken-meat | LWN1492 | Guangzhou |
| 258 | coriander | SYJ580 | Shengyang | 302 | roast chicken | HKJ2436 | Haikou | 346 | frozen chicken-meat | LWN1493 | Guangzhou |
| 259 | roast duck | LWN1484 | Guangzhou | 303 | brine-soaked duck | HKJ2437 | Haikou | 347 | frozen chicken-meat | LWN1494 | Guangzhou |
| 260 | cold noodles in sauce | LWN1489 | Guangzhou | 304 | brine-soaked chicken | HKJ2438 | Haikou | 348 | frozen chicken-meat | PYN1542 | Guangzhou |
| 261 | cold noodles in sauce | PYC1509 | Guangzhou | 305 | brine-soaked pork | HKJ2439 | Haikou | 349 | frozen chicken-meat | ZCJ1643 | Guangzhou |
| 262 | cold vegetable dish in sauce | PYC1510 | Guangzhou | 306 | brine-soaked duck | HKC2411 | Haikou | 350 | frozen chicken-meat | ZCJ1644 | Guangzhou |
| 263 | brine-soaked chicken | PYC1511 | Guangzhou | 307 | brine-soaked chicken | SYC2461 | Shengyang | 351 | frozen chicken-meat | ZJJ1795 | Zhanjiang |
| 264 | roast duck | PYN1534 | Guangzhou | 308 | roast duck | SYJ2484 | Shengyang | 352 | frozen chicken-meat | SGJ1845 | Shaoguang |
| 265 | roast duck | PYN1539 | Guangzhou | 309 | brine-soaked pork | SYJ2485 | Shengyang | 353 | frozen chicken-meat | HYJ1893 | Heyuan |
| 266 | brine-soaked duck | CHJ1587 | Guangzhou | 310 | roast duck | SYJ2487 | Shengyang | 354 | frozen chicken-meat | HYJ1894 | Heyuan |
| 267 | brine-soaked pork | ZCJ1636 | Guangzhou | 311 | brine-soaked duck | SYJ2488 | Shengyang | 355 | frozen pasta | HEBC1968 | Haerbin |
| 268 | brine-soaked pork | SZJ1685 | Shenzhen | 312 | cold vegetable dish in sauce | CDC2559 | Chengdou | 356 | frozen chicken-meat | HEBJ1993 | Haerbin |
| 269 | roast duck | ZJJ1785 | Zhanjiang | 313 | roast duck | CDJ2587 | Chengdou | 357 | frozen chicken-meat | JNJ2032 | Jinan |
| 270 | brine-soaked chicken | ZJJ1788 | Zhanjiang | 314 | brine-soaked duck | WHC2662 | Wuhan | 358 | frozen chicken-meat | BJJ2093 | Beijing |
| 271 | cold noodles in sauce | SGC1810 | Shaoguang | 315 | roast duck | WHJ2684 | Wuhan | 359 | frozen beef | JNJ2033 | Jinan |
| 272 | brine-soaked chicken | SGJ1839 | Shaoguang | 316 | roast duck | WHJ2689 | Wuhan | 360 | frozen chicken-meat | TYJ2142 | Taiyuan |
| 273 | roast pork | HYJ1888 | Heyuan | 317 | brine-soaked chicken | SHJ2734 | Shaihai | 361 | frozen chicken-meat | TYJ2143 | Taiyuan |
| 274 | cold vegetable dish in sauce | HEBC1959 | Haerbin | 318 | brine-soaked duck | SHJ2735 | Shaihai | 362 | frozen chicken-meat | TYJ2144 | Taiyuan |
| 275 | cold noodles in sauce | HEBC1960 | Haerbin | 319 | brine-soaked pork | SHJ2736 | Shaihai | 363 | frozen sheep-meat | TYJ2145 | Taiyuan |
| 276 | cold noodles in sauce | HEBC1962 | Haerbin | 320 | brine-soaked pork | KMJ2786 | Kunming | 364 | frozen chicken-meat | LZJ2182 | Lanzhou |
| 277 | cold noodles in sauce | HEBC1967 | Haerbin | 321 | brine-soaked pork | PYC102 | Guangzhou | 365 | frozen sheep-meat | LZJ2183 | Lanzhou |
| 278 | cold noodles in sauce | PYC1516 | Guangzhou | 322 | brine-soaked pork | PYJ119 | Guangzhou | 366 | frozen chicken-meat | LZJ2197 | Lanzhou |
| 279 | roast pork | JNJ2043 | Jinan | 323 | roast chicken | CHJ177 | Guangzhou | 367 | frozen pork | FZJ2239 | Fuzhou |
| 280 | cold noodles in sauce | BJC2059 | Beijing | 324 | roast duck | SGN341 | Shaoguang | 368 | frozen chicken-meat | FZJ2242 | Fuzhou |
| 281 | cold noodles in sauce | TYC2110 | Taiyuan | 325 | brine-soaked pork | HKN542 | Kunming | 369 | frozen chicken-meat | FZJ2243 | Fuzhou |
| 282 | cold noodles in sauce | TYC211 | Taiyuan | 326 | cold vegetable dish in sauce | SYC559 | Shengyang | 370 | frozen sheep-meat | FZJ2245 | Fuzhou |
| 283 | brine-soaked pork | BJJ2087 | Beijing | 327 | roast duck | SYJ 577 | Shengyang | 371 | frozen chicken-meat | NNJ2293 | Nanchang |
| 284 | brine-soaked pork | TYJ2135 | Taiyuan | 328 | cold vegetable dish in sauce | FZC709 | Fuzhou | 372 | frozen chicken-meat | NNJ2294 | Nanchang |
| 285 | brine-soaked beef | TYJ2136 | Taiyuan | 329 | cold vegetable dish in sauce | HEBC1159 | Haerbin | 373 | frozen chicken-meat | XMJ2344 | Xiamen |
| 286 | brine-soaked beef | TYJ2137 | Taiyuan | 330 | milk | TYJ2133 | Taiyuan | 374 | frozen chicken-meat | XM266 | Xiamen |
| 287 | brine-soaked pork | LZC2159 | Lanzhou | 331 | frozen pasta | XAC1216 | Xi’an | 375 | frozen chicken-meat | HKJ2443 | Haikou |
| 376 | frozen chicken-meat | HKJ2444 | Haikou | 394 | frozen pasta | CHC153 | Guangzhou | 412 | Pleurotus eryngii | LZC2171 | Lanzhou |
| 377 | frozen sheep-meat | HKJ2445 | Haikou | 395 | frozen pasta | SYC565 | Shengyang | 413 | needle mushroom | LZJ2199 | Lanzhou |
| 378 | frozen sheep-meat | SYJ2495 | Shengyang | 396 | frozen chicken-meat | SYJ581 | Shengyang | 414 | needle mushroom | FZC2219 | Fuzhou |
| 379 | frozen chicken-meat | CDJ2593 | Chengdou | 397 | frozen sheep-meat | SYJ582 | Shengyang | 415 | needle mushroom | FZJ2247 | Fuzhou |
| 380 | frozen chicken-meat | CDJ2594 | Chengdou | 398 | frozen chicken-meat | NNC667 | Nanchang | 416 | Pleurotus eryngii | FZJ2248 | Fuzhou |
| 381 | frozen chicken-meat | CDJ2595 | Chengdou | 399 | frozen pasta | FZC715 | Fuzhou | 417 | oyster mushroon | FZJ2249 | Fuzhou |
| 382 | frozen chicken-meat | HFJ2642 | Hefei | 400 | frozen pork | HFJ895 | Hefei | 418 | needle mushroom | NNJ2299 | Nanchang |
| 383 | frozen chicken-meat | HFJ2644 | Hefei | 401 | white beech mushroom | PYN1549 | Guangzhou | 419 | needle mushroom | BHC2369 | Beihai |
| 384 | frozen chicken-meat | HF307 | Hefei | 402 | shiitake mushroom | ZCC1621 | Guangzhou | 420 | needle mushroom | HKJ2446 | Haikou |
| 385 | frozen chicken-meat | HF312 | Hefei | 403 | oyster mushroon | ZCJ1648 | Guangzhou | 421 | needle mushroom | HKJ2449 | Haikou |
| 386 | frozen chicken-meat | WHJ2693 | Wuhan | 404 | oyster mushroon | ZCJ1649 | Guangzhou | 422 | needle mushroom | SYJ2497 | Shengyang |
| 387 | frozen chicken-meat | SHJ2742 | Shaihai | 405 | straw mushroom | ZCJ1650 | Guangzhou | 423 | needle mushroom | CDC2570 | Chengdou |
| 388 | frozen chicken-meat | SHJ2743 | Shaihai | 406 | straw mushroom | CHJ1600 | Guangzhou | 424 | white beech mushroom | WHJ2699 | Wuhan |
| 389 | frozen chicken-meat | SHJ2745 | Shaihai | 407 | straw mushroom | ZJJ1800 | Zhanjiang | 425 | shiitake mushroom | SHC2720 | Shaihai |
| 390 | frozen pasta | YXC004 | Guangzhou | 408 | straw mushroom | HYJ1900 | Heyuan | 426 | oyster mushroon | SHJ2749 | Shaihai |
| 391 | frozen chicken-meat | PYJ121 | Guangzhou | 409 | oyster mushroon | XAJ1950 | Xi’an | 427 | needle mushroom | ZJJ383 | Zhanjiang |
| 392 | frozen pork | HKC516 | Haikou | 410 | needle mushroom | BJC2069 | Beijing | 428 | white beech mushroom | NNC669 | Nanchang |
| 393 | frozen sheep-meat | HKC 517 | Haikou | 411 | needle mushroom | BJJ2097 | Beijing | 429 | needle mushroom | NNN698 | Nanchang |

Table S2 Sequences of primers used in paper.

| Genes | Primer sequence (5’- 3’) | Ampliconsize  (bp) | PCR conditions (°C, S)* | | | Reference |
| --- | --- | --- | --- | --- | --- | --- |
|  |  |  | Denaturation | Annealing | Extension |  |
| CTX-M | F: ATGTGCAGYACCAGTAARGTKATGGC | 592 | 94, 60 | 55, 60 | 72, 60 | Dierikx et al., 2012 |
|  | R: TGGGTRAARTARGTSACCAGAAYSAGCGG |  |  |  |  |  |
| SHV | F: TTATCTCCCTGTTAGCCACC | 796 | 94, 60 | 55, 60 | 72, 60 | Dierikx et al., 2012 |
|  | R: GATTTGCTGATTTCGCTCGG |  |  |  |  |  |
| TEM | F: GCGGAACCCCTATTTG | 964 | 94, 60 | 55, 60 | 72, 120 | Dierikx et al., 2012 |
|  | R: ACCATTGCTTAATCAGTGAG |  |  |  |  |  |
| OXA | F: ACACAATACATATCAACTTCGC | 813 | 94, 60 | 61, 60 | 72, 90 | Sa´enz et al., 2004 |
|  | R: AGTGTGTTTAGAATGGTGATC |  |  |  |  |  |
| IntⅠ | F: GGGTCAAGGATCTGGATTTCG | 483 | 94, 60 | 62, 60 | 72, 60 | Sa´enz et al., 2004 |
|  | R: ACATGGGTGTAAATCATCGTC |  |  |  |  |  |
| IntⅡ | F: CACGGATATGCGACAAAAAGGT | 788 | 94, 60 | 62, 60 | 72, 60 | Sa´enz et al., 2004 |
|  | R: GTAGCAAACGAGTGACGAAATG |  |  |  |  |  |

*: PCRs were performed with an initial denaturation step of 94 °C for 5 min, 35 cycles each of denaturation, annealing and extension as indicated and a final extension of 10 min at 72 °C

References used in Table S2

Dierikx C.M., van Duijkeren E., Schoormans A.H.W., van Essen-Zandbergen A., Veldma K., et al. Occurrence and characteristics of extended-spectrum-b-lactamase and AmpC-producing clinical isolates derived from companion animals and horses. J Antimicrob Chemother, 2012, 67: 1368-1374.

Sa´enz Y., Brin´as L., Domínguez E., Ruiz J., Zarazaga M., et al. Mechanisms of Resistance in Multiple-Antibiotic-Resistant Escherichia coli Strains of Human, Animal, and Food Origins. Antimicrobial Agents and Chemotherapy, 2004: 3996-4001.
